# Coupling Wearable Devices and Decision Theory in the United States Emergency Department Triage Process: A Narrative Review

**DOI:** 10.3390/ijerph17249561

**Published:** 2020-12-21

**Authors:** Valentina Nino, David Claudio, Christie Schiel, Brendan Bellows

**Affiliations:** Mechanical & Industrial Engineering Department, Montana State University, Bozeman, MT 59715 USA; cl.schiel@gmail.com (C.S.); brendan.bellows@gmail.com (B.B.)

**Keywords:** MAUT, continuous patient monitoring, emergency department, patient prioritization, closed-loop system

## Abstract

This research was motivated by the nurses’ decision-making process in the current emergency department (ED) triage process in the United States. It explores how continuous vital signs monitoring can be integrated into the ED. The article presents four shortcomings on current ED triage systems and proposes a new conceptual clinical decision support model that exploits the benefits of combining wireless wearable devices with Multi-Attribute Utility Theory to address those shortcomings. A literature review was conducted using various engineering and medical research databases, analyzing current practices and identifying potential improvement opportunities. The results from the literature review show that advancements in wireless wearable devices provide opportunities to enhance current ED processes by monitoring patients while they wait after triage and, therefore, reduce the risk of an adverse event. A dynamic mathematical decision support model to prioritize patients is presented, creating a feedback loop in the ED. The coupling of wearable devices (to collect data) with decision theory (to synthesize and organize the information) can assist in reducing sources of uncertainty inherent to ED systems. The authors also address the feasibility of the proposed conceptual model.

## 1. Introduction

Emergency departments (EDs) handle high quantities of patients every day, each with varying criticality and needs. Patient triage is used to manage a large volume of patients and sort them based on the severity of their condition [1,2]. Various types of triage systems can be used to sort patients waiting in the ED [3].

EDs in countries such as Australia and Canada have developed their five-level triage system. For example, Australia uses the Australasian (National) Triage Scale [4], Canada has the Canadian Triage and Acuity Scale (CTAS) [5], and the UK implements the Manchester Triage system [6]. Some EDs in the United States (US) currently use a three-level system. Over the last decade, five-level systems have gained acceptance, as researchers have demonstrated the effectiveness and reliability compared to the three-level system [5,7,8,9].

The Emergency Severity Index (ESI) is a common example of a five-level triage system. ESI is used by ED nurses at many hospitals in the US to prioritize patients and sort them into five distinct levels [7]. Once an ESI triage level is assigned, a patient can be directed to registration, initial treatment, or waiting, based on their acuity and presumed resource needs. A patient’s ESI level determines how quickly they will be treated; for instance, a patient with an ESI of 1 may be given immediate treatment, while a level 4 patient may have to wait before being admitted [10].

**Figure 1:**
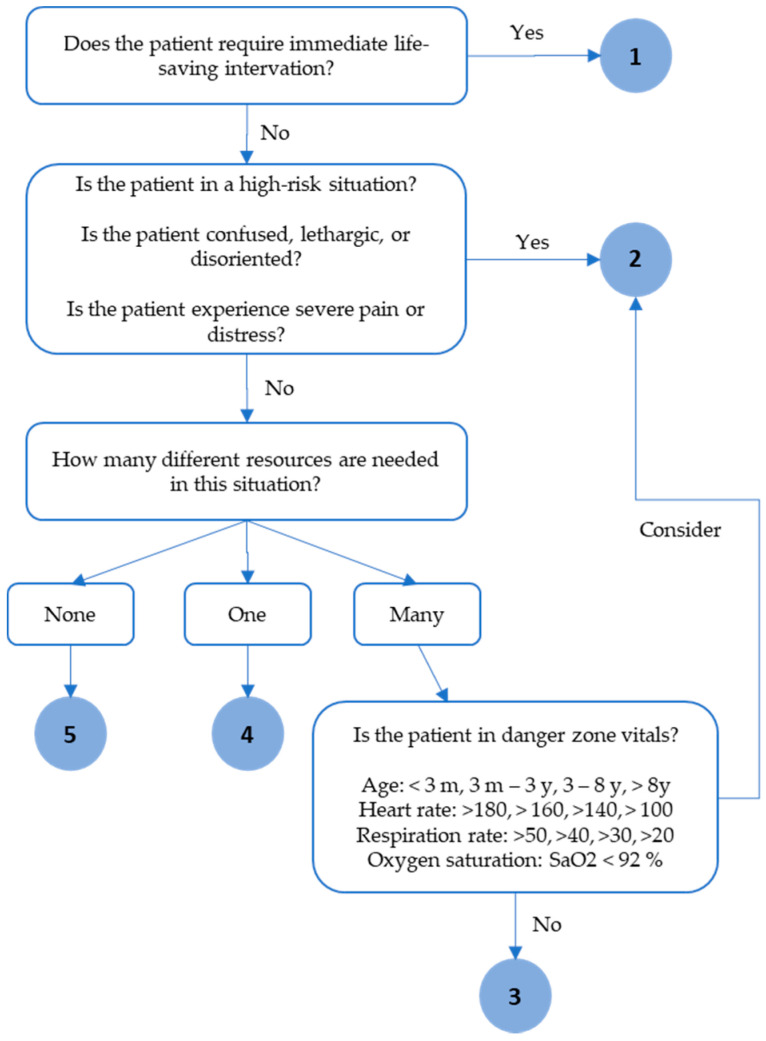
ESI Triage Algorithm (Gilboy et al., 2005).

The patient’s vital signs, blood pressure, pulse, temperature, respiration rate, and oxygen saturation are assessed to classify ESI levels. Other factors taken into consideration include present illness, allergies, and past medical history [2]. Figure 1 displays the fourth revision of the ESI Triage Algorithm, constructed by Gilboy et al. (2005) [11].

According to Figure 1, when a nurse identifies a patient who requires immediate intervention, the patient is assigned an index value of 1. High-risk situations are assigned an index value of 2. However, there is some debate for people who fall on the borderline between indices 2 and 3. According to the flowchart, vital signs play an important role in this decision.

ESI has successfully improved consistency and accuracy in ED prioritization [12]; however, there are still shortcomings of the ESI system used in the US, some of which include: (I) once a patient is assigned to an ESI level, their condition is not monitored, therefore leaving a chance that patient deterioration will go unnoticed by the ED nurse [13]; (II) triage level assessment is a subjective process based on the ESI standards, it incorporates a large portion of the individual nurse experience as well as uncertainty [14]; (III) when patients are classified within the same triage level, they are admitted on a first-come-first-serve basis (FCFS), which may not entirely reflect the level of severity of each patient’s condition [13]; (IV) nurses are not given feedback on the outcome of their prioritization decisions; therefore, they do not have evidence that they should follow similar courses of action, or change their criteria for subsequent decisions in similar situations.

This article explores these shortcomings and explores the use of wearable devices to continuously monitor patients’ vital signs in the ED to generate a dynamic mathematical decision support model to prioritize patients. The proposed conceptual model will provide an opportunity to create a feedback loop to help nurses learn about the outcomes of the decisions they make and apply this knowledge to future decision making.

## 2. Materials and Methods

A review of the literature was conducted to identify the shortcomings of current US triage processes and potential improvement opportunities. The topics and keywords explored include patient prioritization, patient monitoring, wearable devices, nurse workload and feedback, triage assessment processes, and patient prioritization systems. The literature review also compiles information regarding the feasibility of the proposed conceptual model, including information that can be carried forward in the proposed research and additional areas that can be further explored.

### 2.1. Shortcomings of the US Triage Process

#### 2.1.1. Patient Monitoring

In the US, a patient’s condition is usually no longer monitored once they are assigned to an ESI level, which means the ED nurse may not be aware of patient status deterioration. Monitoring a patient’s vital signs through wearable devices can alert nurses to deteriorating conditions. Ashour and Kremer indicate that a patient’s status can change over time, and therefore, the triage system should incorporate these changes [2].

A study by Johnson et al. found that approximately 15% of patients recorded critically abnormal vital sign values [10]. This highlights the importance that vital sign monitoring has on detecting critical conditions in a significant percentage of patients. Johnson et al. also found that 62% of patients documented abnormal vital signs; however, few of these abnormalities significantly increased the patient’s overall criticality enough to signal a nurse for intervention [10]. While wearable devices can provide immediate notification of abnormalities, it is important to consider the overall weight of the abnormal vital sign relative to other signals. Descriptive information, such as age, gender, past medical history, and complaint, in conjunction with vital signs, including pulse, respiration rate, and oxygen saturation, can provide a good prediction of a patient’s overall risk of deterioration [13]. Monitoring these patient attributes and tracking patient conditions with wearable devices provides an opportunity to alert nurses when patient conditions become critical. Furthermore, this information could be used to supplement the ESI in prioritizing patients [13].

Dugas et al. [1] indicate that almost half of the patients are assigned to an ESI level 3, highlighting that ESI does not adequately distribute patients across the five levels. The result can delay treatment to those that need immediate care because other patients with varying criticality are clustered as equals into one ESI level [2]. While the ESI is a helpful tool to differentiate between patient needs, it is ultimately sorting patients into discrete intervals and assigning equivalent weights to very different patient statuses.

#### 2.1.2. Subjective Triage Assessment and Uncertainty

A common complaint of the US ESI prioritization method is its dependence on user preference. Dugas et al. indicate ESI prioritization incorporates subjectivity during triage, and systematic flaws in triage assessment are possible [1]. Additionally, the ESI system may be limited by variability from nurse inexperience and human error [1]. For example, Johnson et al. found that the patient’s consciousness level had the most significant effect on triggering a critically abnormal composite score, and level of consciousness is the only subjective vital sign within this score [10]. This highlights that a nurse’s subjective reasoning has the power to significantly impact patient prioritization.

In addition to subjectivity in assessing patient conditions, Ryynänen et al. [15] conducted a study and found that unintentional bias can also impact a nurse’s prioritization decisions. Implicit bias is an unconscious mental model of certain social groups. It is unintentional and difficult to detect. Studies have found that, even when people identify themselves as nonprejudiced, egalitarian, and nondiscriminatory, they may still display implicit bias in their attitudes and actions [15]. The implications of this in the prioritization of patients can be critical, as nurses may unknowingly incorporate bias against certain patient demographics in their decisions.

Dong et al. address an additional problem with the ESI method, indicating that it relies too heavily on memory and that nurses should neither have to reference the paper version of the triage nor recall its contents from memory [16]. Additionally, lack of time, triage complexity, and recall bias can make it difficult for nurses to prioritize patients, increasing the subjectivity of decisions and leading to inconsistency in the triage process [16].

Another of the biggest challenges nurses face at the triage is uncertainty. Uncertainty is typically credited to lack of understanding, imperfect or incomplete information, lack of experience, the inability to predict the future with certainty, or not having or understanding preference between alternatives.

Løken et al. categorized uncertainty as either external or internal. External uncertainties denote circumstances that are not controllable by the decision-maker (DM) and might influence the decision outcome. Internal uncertainties are related to the DM’s preferences [17] and are caused primarily by people being unsure about which option best reflects their preferences. For example, in ED settings, the exact diagnosis of a patient cannot be known with certainty at the early stages of the process (i.e., triage). For this reason, nurses and physicians must make a choice that involves risk [18]. The external uncertainty is on how the patient’s condition will progress as he/she waits or the effect of a treatment on a patient. The internal uncertainty is of the nurse’s unclear choice of which patient has a higher priority when several patients are waiting with different complaints, attributes, and symptoms.

#### 2.1.3. First-Come-First-Serve Prioritization within Groups

Williams found that roughly one-half of patient visits to the ED are urgent. The problem is that there is a vast disparity in the classification of the other patients between non-urgent and semi-urgent categories [19]. There might be cases when several patients waiting for care have the same priority (i.e., several patients with ESI level 3). In these cases, a nurse needs to decide which patient needs care first. When several patients have the same urgency, it forces nurses to use additional knowledge to discern the priority [20].

In many cases, nurses use a first-come-first-serve (FCFS) rule for patient selection when several patients have the same priority and are waiting in the ED after triage. This disregards the fact that the patient’s status might improve or degrade while waiting. Prioritizing on an FCFS basis may overlook a patient that has a condition worse than someone who arrived earlier but was assigned to the same triage level [13].

Current CDSS applications assist nurses with patient prioritization when they are evaluated at the triage. However, there is limited research on nurses’ decision-making process after patients have gone through the triage. Besides, current algorithms evaluate one patient at an individual level and then place the patient in a group rather than evaluating all patient priority at the same time. Claudio et al. [21] argued that statistical predictive models could be used to assign a distinct priority number to each patient, which eliminates the necessity of sorting patients into groups or categories. Ultimately, the reasoning behind a CDSS is that patients within each ESI level will also be sorted and prioritized based on vital signs that are continuously collected through wearable devices along with their descriptive information. By eliminating categorization and providing distinct prioritization, nurses no longer have to use FCFS to determine which patients should receive care first. An additional benefit of eliminating this step is that nurses might be able to reduce the time it takes them to make the assessment and, thus, reduce the total triage time.

#### 2.1.4. Nurse Decision Making and Lack of Feedback

Several studies have looked at nurses’ decision-making process in the ED and how these decisions affect patient prioritization. These studies have found that nurse decision making is a skill that is based on pattern recognition, including heuristics and rules of thumb, which are problem-solving techniques that are mental shortcuts to help in making decisions based on practice and experience [22]. This indicates that nurses with more experience may have a better skill set to prioritize patients than novice nurses.

Studies on nurses’ decision-making processes have found that much of the decision making was based on the nurse’s experience [23], knowledge, and intuition [24,25,26]. Pott et al. argue that, in dynamic situations, a decision maker (DM) will look for the expected response, according to familiar situations [27]. The DM looks for cues, which will let him/her compare the current situation with similar past situations. If the DM can match the current situation with previous ones, then he/she proceeds to act similar to a previous time. In unfamiliar situations, deliberations are necessary, meaning that the DM will have to either identify a familiar situation in an unfamiliar setting or create a new solution. Pott et al. named these processes “mental simulation” and “story building” [27].

The idea that both new and experienced nurses rely on heuristics to make decisions presents a key challenge with the ESI and EDs. Within triage, there is no prompt and direct feedback loop to inform nurses of the outcomes of their decisions. Nurses do not receive feedback regarding the patient’s subsequent treatment and the end result of their visit. A system that does not include feedback is considered an open-loop system. A challenge with an open-loop system is that processes are not refined based on outcomes and ultimately lead to an end result that might deviate from the desired outcome. Therefore, the rules of thumb and heuristic approaches that nurses developed are based on only what is seen in triage and lack key information about the outcome of a patient’s visit. Subsequently, nurses are making decisions based on these heuristics that do not incorporate the overall treatment of a patient. The proposed conceptual model can address this omission of feedback to nurses since it will be designed as a closed-loop system. A closed-loop system incorporates feedback, such that the process is continually modified and improved based on previous outcomes.

**Figure 2:**
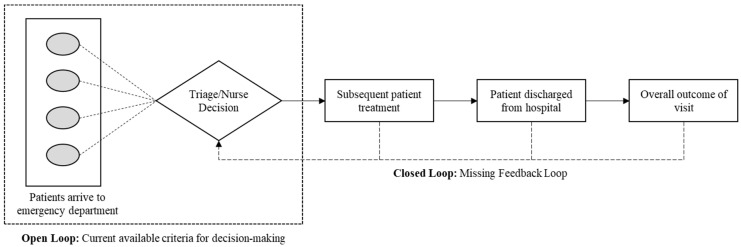
US Emergency department feedback loop.

Figure 2 displays the contrast between a closed-loop and an open-loop system; it highlights the difference in the amount of available information for nurses to base triage decisions in an open-loop versus a closed-loop system. It is important to recognize that when nurses make decisions, one or more desirable outcomes are possible [22]. Additionally, there is no general clinical decision-making method for nurse decision-making, but it varies greatly from person to person [22]. Another consideration that affects the feedback on decision-making is the complexity of prioritizing multiple patients and the interaction effects between these different patient scenarios. A patient’s priority is relative to other patients’ status, and while certain criteria may increase their importance in one instance, they may be viewed as less critical in another.

As seen in Figure 2, structuring a feedback loop to aid nurses in decision-making must tailor to these considerations and ultimately help develop appropriate heuristics. This can even expedite the learning curve such that a novice nurse can enhance their skills and more quickly be comparable to a nurse with years of experience [28]. Ultimately feedback in the ED should be frequent and quantifiable, ensuring information is presented in a meaningful and concise way while still relevant to the nurses.

Current feedback in US EDs is often provided in the form of a standardized peer-review process that identifies and analyzes medical errors in the ED [29]. In general, the peer-review process begins by having the faculty review incident report cases. When this review indicates a potential medical or system error, the case is further reviewed by a peer review committee [30]. The review committee studies the facts of the case and determines if an error occurred [30]. Errors can occur on the basis of taking an incorrect action or failure to take action.

Peer review is a feedback process that highlights areas of potential process improvements and can increase a nurse’s knowledge and enhance patient care [31]. A major challenge with the peer-review process is that there is no clear and defined review methodology standardized for widespread use [29,31]. Additionally, peer review is not used in all nursing organizations, and it is typically only used annually [31,32]. The peer-review process is often implemented during annual performance evaluations or when cases are referred by nurses, risk management, or review committees due to potential medical errors [31].

This highlights two gaps in the peer review feedback loop: (1) feedback is not being provided promptly, and the frequency nurses receive it is minimal; (2) feedback is only provided based on cases that are referred to the peer-review committee due to potential medical errors. While the peer-review process provides a means to communicate evaluations to nurses [33], there is an opportunity to give nurses feedback on decisions that are not necessarily considered “medical errors” but can still impact a patient’s ED visit outcome, specifically regarding their patient prioritization decisions. Additionally, providing feedback more frequently can improve the learning process for nurses to base on future decisions.

### 2.2. Continuous Patient Monitoring

Patient monitoring devices and applications have been developed and tested in numerous frameworks, which include combat casualty care [34], emergency response to mass casualty incidents [35,36,37,38,39], hospital operations management [40,41,42], and telemedicine [43,44,45,46,47,48].

**Figure 3:**
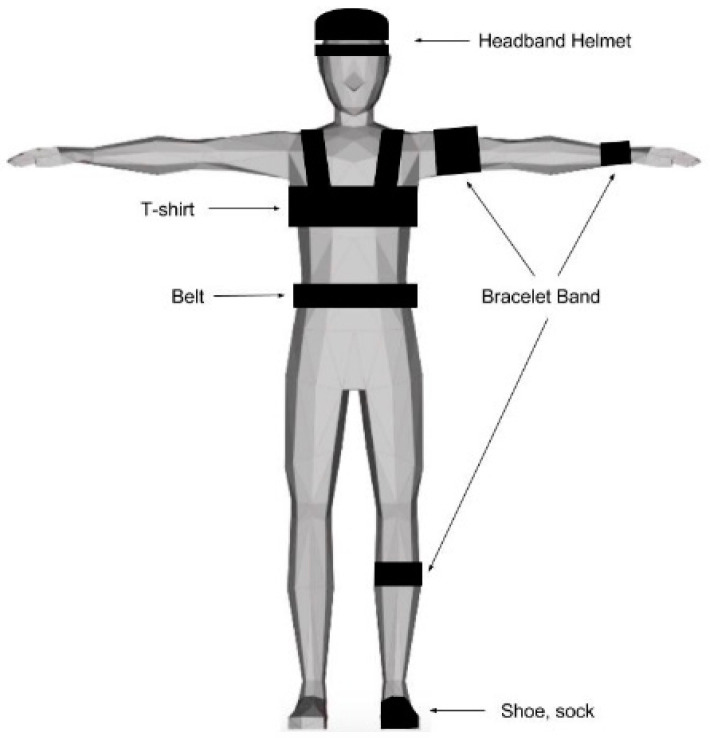
Main locations of biomedical sensors for ambulatory uses [49].

The initially reported cases were for military applications [34] and patient tracking in emergency response situations. In recent years, researchers have proposed combining biomedical instruments with wireless technology to track, categorize, and monitor patients along with their vital signs. Gao et al. (2005) conducted a study to determine the vital signs that are good candidates to monitor based on protocols and discussions with paramedics [39]. According to Gao et al. (2005), candidate vital signs are blood pressure, pulse, temperature, respiration rate, oxygen saturation, peripheral vascular perfusion, mental status, and electrocardiography. Furthermore, a survey to assess the importance of the candidate’s vital signs to user needs reveals that pulse was the most important vital sign to measure, followed by oxygen saturation and blood pressure. According to Dittmar et al. (2005), several areas can be used for biomedical sensing, obtaining the best signal/noise ratio, the best fixing, and the best ergonomics, but also discrete and painless [49]. These areas are illustrated in Figure 3. According to the figure, the best way to implement biomedical sensors is by using headbands, t-shirts, belts, and bracelet bands.

Curtis et al. (2008) were among the first ones who proposed using biosensors in the Emergency Department (ED) on everyday operations. They developed the Scalable Medical Alert Response Technology (SMART) system with the purpose of using wireless technology to constantly monitor patients’ vital signs [41]. SMART enables the continuous monitoring of patients after they are triaged. The primary application for this technology is for disaster relief [41]. The system integrates wireless patient monitoring, geo-positioning, signal processing, and targeted alerting to monitor patient status and location and can also be applied to overcrowded EDs [41].

While this system continuously monitors patient conditions, its primary use is to not prioritize patients. The SMART system only affects patient prioritization when vital signs trigger an alarm that the patient’s condition is serious enough to reassign priority.

A prototype of the SMART system was created and implemented in a pilot study in an ED waiting room. The development of this prototype provided useful considerations in the implementation of a continuous patient monitoring system, including the fact that these types of sensors must be cost-effective, accurate, have the capability to communicate data while ensuring privacy/confidentially, and could be used for early diagnosis or to facilitate experts’ diagnostics that allow for protecting patients against further risks [41,50,51,52]. In addition, physical sensor limitations, such as battery life, power source, sensor durability, easy-to-use, and carrying case impact system use implementation [51]. Furthermore, considerations should include an alert system, conditions that trigger alarms, false positives, and alarm fatigue [52].

### 2.3. Wearable Devices

A variety of sensors and wearable devices to monitor vital signs are on the market or in development. These devices offer varying capabilities and functions. Table 1 provides basic device information based on a previous study by Pantelopoulos and Bourbakis [53].

A variety of wireless devices and sensors can be used to obtain continuous vital signs information from patients in the ED. These devices can be evaluated using metrics such as cost, placement (invasiveness), durability, reusability, reliability, communication capabilities, and integration abilities [53].

It can be seen that most of the research done with the technology serves the purpose of data collection, and only a few have proposed a prioritization model using the data obtained. Kohli and Piontek (2008) [54] point out that advances in information technologies create new opportunities for addressing healthcare challenges.

Table 2 shows the capabilities of the different devices to measure, collect, and transmit information of the most typically vital signs collected at triage. According to the table, only a few of these devices collect blood pressure, heart rate, pulse oximeter, respiration, and temperature altogether. Only six of them collect blood pressure automatically.

### 2.4. Patient Prioritization Systems

Existing research that addresses patient prioritization through triage algorithms includes the DGP Triage Algorithm [2], eTriage [16], Electronic Triage System [1], and Soterion Rapid Triage System [28].

DGP Triage Algorithm [2]: Group technology (GT) is used to cluster patient attributes into groups based on patient characteristics, such as chief complaint, gender, age, vital signs, expected treatment time, or use of laboratory tests. The DGP (dynamic patient grouping and prioritization) triage algorithm is then used to prioritize patients within each of these groups.

The algorithm incorporates vital signs, including temperature, pulse, respiration rate, and blood pressure, in addition to age, gender, and pain level. The DGP algorithm is solved iteratively; consequently, patients are continually being evaluated against one another. This algorithm differentiates between patients within the same ESI triage level by generating an overall prioritization score. An additional outcome is that the DGP algorithm shortens the patients’ average length of stay, the average time to bed, and time in the ED compared to the standard ESI method.

Although the DGP algorithm’s final output is distinct prioritization between patients, it still clusters patients based on attributes prior to generating an overall prioritization score. Once patients have been clustered, the DGP might not allow patients to move between groups. Research involving the DGP algorithm does not address continuous vital signs monitoring and changing patient conditions. It instead uses “available and recorded” triage data, which may not accurately reflect patient status.

*eTriage* [16]: This is a web-based triage support system used with the Canadian Triage and Acuity Scale (CTAS) in the ED to reduce variability and standardize triage assessment. A nurse chooses from a standardized patient complaint, eTriage then displays a CTAS template. The template is specific to the indicated complaint and helps assign the appropriate triage level. When the nurse generates a score that differs from the score generated by eTriage, they can give an explanation to override the system. Nurses are encouraged to override the system based on clinical judgment because these overrides can be used to adjust the eTriage criteria. The eTriage system’s outcome is a discrete categorization of patients that do not differentiate between patients given the same triage score.

*Electronic Triage System (ETS)* [1]: ETS is used to separate ED patients into triage levels. The system is based on the risk of critical or time-sensitive patient outcomes, including mortality, admission to the ICU, or direct transport to the OR. Logistic regression is used to calculate this probability and is based on vital signs, demographics, primary chief complaint, and how the patient arrived at the ED. The probability of the patient outcome is then used to assign the patient to one of the five ETS triage levels. ETS decreases subjectivity in prioritization and found key factors that increase the probability of a patient having a critical outcome include higher age, arrival by ambulance, abnormal vital signs, and specific chief complaints, such as chest pain, abdominal pain, shortness of breath, and fever/chills.

Although ETS creates a better distribution of patients across the five triage levels, the outcome is a discrete categorization of patients that do not differentiate between patients given the same triage score. Additionally, ETS cannot account for critical details that a nurse can identify and assign, such as patient history or appearance. Therefore, the nurse cannot override the ETS score based on their concerns, specific injuries or illnesses, or resource availability.

*Soterion Rapid Triage System (SRTS)* [28]: SRTS is a 5-level computerized triage system based on patient vital signs, general observations of the patient, and a complaint-based algorithm. The system incorporates two separate algorithms for adults and children, and its measurable outcomes include ED length of stay, resource consumption, Procedural Terminology Codes, and in-hospital admission rate. A potential shortcoming of the SRTS is that it does not differentiate between patients assigned to the same level within the triage system. Additionally, the algorithms used in this system are based on vital signs taken when the patient first arrives at the ED, neglecting potential changes in patients’ conditions. This highlights the opportunity to create a robust triage prioritization that improves efficiencies, such as the SRTS and incorporates continuous vital signs monitoring and prioritizes between patients assigned within the same triage level.

Previous research in patient prioritization highlights the need for classification within triage levels. The DGP triage algorithm is the only existing method that prioritizes patients within triage levels; however, it still clusters patients during the prioritization process. Moreover, none of these studies integrated wearable devices for continuous patient vital signs monitoring. Therefore, prioritization decisions do not reflect changes in patients’ conditions, and critical vital signs may be overlooked. Using wearable devices to monitor vital signs continuously can create an opportunity to expand on these already-existing prioritization methods and provide information about changing patient conditions.

## 3. Results

### 3.1. Proposed Clinical Decision Support Model

US Emergency Departments (EDs) use triage systems to sort patients by severity of illness or injury [5,24,78,79,80]. Current triage systems or algorithms are based on the nurse’s initial triage, which could include assessing patient vital signs when patients arrive at the ED. Healthcare researchers have suggested improvements to the current triage system [81,82]; however, a major drawback continues to resolve around its dependency on the vital indicators originally measured by ED nurses and not followed sequentially over the usually critical waiting period. Therefore, a clinical decision support system (CDSS) that incorporates continuous vital signs monitoring (captured through wearable devices) and descriptive information to detect changes in patient conditions and identify patients with deteriorating conditions is needed.

The Institute of Medicine published a milestone report describing overcrowding crises in EDs in our nation [83]. There are reported cases of patient deaths while waiting in the ED after being assessed at triage [84,85]. It is possible that these deaths could have been prevented by either better assessment during triage or by better patient monitoring [78]. However, this may not be possible in an understaffed or overcrowded ED [86].

Understaffed or overcrowded EDs is one of the main reasons why current triage systems have incorporated neither deterioration nor improvement of vital signs after triage and while patients are waiting, even knowing that when patients must wait after triage, either in the lobby or in an assigned room, there is considerable uncertainty with regard to the changes in vital signs and patient status. To be aware of both, a baseline measure and reassessment of vital signs are needed. The proposed decision support model addresses this gap by integrating mathematical models with continuous monitoring wearable devices.

The proposed dynamic decision support model for patient prioritization in the ED will be based on multi-attribute utility theory (MAUT), fuzzy logic, and the use of decision trees. Multi-attribute utility theory (MAUT) [87] is a methodology that considers uncertainty and the disposition to make trade-offs at several attribute levels. MAUT is a quantitative technique for capturing individuals’ preferences or values [33,88]. It involves a single DM who chooses among several alternatives grounded on two or more criteria or attributes. The alternatives and decision-making context involve risks and uncertainties. The DM seeks to maximize a value function that depends on the attributes [87].

MAUT integrates mathematical concepts and assessment methods that collectively order alternatives in a structured manner and address the decision-making problem [89]. When the DM’s preferences are identified, and a predetermined set of attributes is defined, mathematical functions can be built to assess the attributes according to the preferences [90]. Solving a problem via utility functions helps the DM make an informed and calculated decision that maximizes the DM’s approval. In short, MAUT could be used to aid nurses in their triage assessments.

The coupling of technology and decision theory can reduce the level of uncertainty inherent to triage nurses’ decision-making process. Using decision theory to prioritize patients can reduce the subjectivity of patient prioritization and eliminate bias by providing empirical justification. Wearable devices will be used to collect patients’ vital signs in real-time information, which will then be integrated into mathematical decision models to inform future decision making. The monitoring devices serve the purpose of collecting data, while the mathematical models will be designed to assist nurses in data gathering, analysis sorting, and presentation.

Claudio, Ricondo, Freivalds, and Okundan (2012) [21] investigated the possibility of using patients’ descriptive information, such as age, gender, and complaint, along with vital signs collected at the triage, to predict the ESI of the patients. Their study found that patient complaint, age, pulse, and respiration rate were the best predictors of ESI. Their findings of the vital signs were also consistent with Cuthbertson, Boroujerdi, McKie, Aucott, and Prescott (2007) [91], who also found that in ICU, pulse and respiration rate, along with oxygen saturation, were also good predictors in determining a patient’s risk of health deterioration.

Ashour and Okudan (2013) [92] and Ashour and Okudan (2010) [93] proposed the use of Fuzzy AHP along with utility theory. However, their method was tested via a simulation rather than real data. In addition, they did not validate the values of the preferences of nurses.

Claudio et al. (2014) [13] already tested MAUT for patient prioritization in the ED. The preliminary study results support the hypothesis that the use of decision models to aid nurses in the decision-making process is very close to what a medical expert would suggest. However, the algorithm was updated at discrete points in time, whereas the proposed algorithm updates continuously.

The integration of wearable devices and advanced sensing capabilities provides the potential to establish a data-rich emergency room triage system, which can further improve the quality of care via the continuous monitoring of patients.

#### 3.1.1. Proposed System Architecture

The proposed decision support model consists of a patient monitoring device, a wireless networking system, and a mathematical decision support system. Table 1 and Table 2 provide evidence that the technology regarding monitoring devices to supplement the proposed conceptual model already exists.

After extensive research on current monitoring systems, the wearable monitoring device we decided to use is the CareTaker 4 Wireless Vital Signs Monitor, manufactured by Caretaker Medical a company based in Charlottesville, Virginia, USA [77]. The CareTaker is an FDA-approved wearable device. It is a cardiovascular monitoring device with the advantages of measuring, in a non-invasive and continuous way, blood pressure, heart rate, and respiration rate, as illustrated in Table 2.

**Figure 4:**
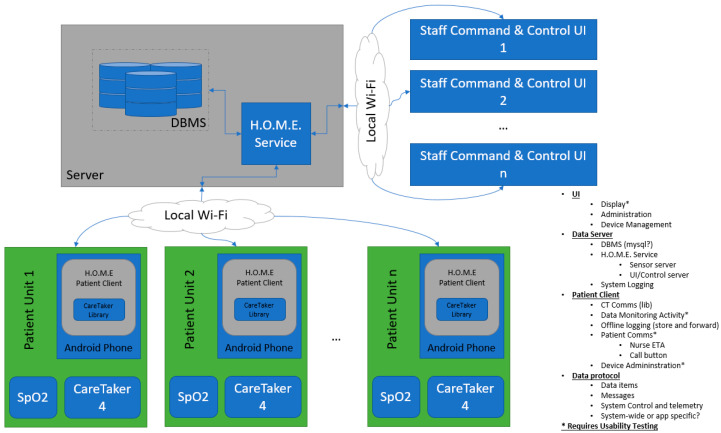
Proposed system architecture.

Figure 4 shows the main components of the Healthcare Ongoing Monitoring Equipment (HOME) system architecture. HOME sensor monitoring implementation is architected to allow for flexibility. On the client side (patient side), a single CareTaker 4 sensor device connects to a gateway app running on an Android device. This connection is maintained over Bluetooth Low Energy (BLE). Additionally, a wireless pulse oximeter connects to the same gateway app to provide pulse information to the system. This unit also connects via BLE. The gateway app collects the information from the devices and submits this to the HOME service running on a networked server device. In early implementations, this has been running on a Raspberry PI or O-Droid single board computer. The service stores these data in a MySQL database, stored on the machine running the service. This server keeps data for postprocessing, historical review, etc.

Healthcare professionals connect to the server using devices that subscribe to updates from the service. Sensor clients publish data to the service as it becomes available. The service then forwards that data to any connected display client that desires the data from that client (patient) while retaining a timestamped copy in the database. Data are exchanged in a JSON message format. In current iterations of the system, communications take place over dedicated TCP sockets. For future versions, the system will move to a more extensible and secure format, likely implementing a REST API to maximize ease of integration with other test systems.

The admin/healthcare interface is currently written in C++ and runs on a Windows tablet with a touchscreen. It is designed to be lightweight so that it can be deployed on low power and low-cost systems if needed. An additional UI will be developed for iOS to run iPad devices. Current networking equipment uses Ubiquiti UniFi access points with a Ubiquiti EdgeRouter (from Ubiquiti Inc., New York, NY, USA) for security, traffic segregation, and monitoring. This allows the system to provide secure connections between the devices without requiring additional licensing or interfering with other equipment in the test environment.

#### 3.1.2. Proposed Mathematical Decision Model

The development of the proposed mathematical decision support model initiates with analyzing historical data collected from visits to the ED to develop mathematical models for nurses’ decision making in the ED. The data are used as input to classification tree analysis to identify patterns and dependence in the data, which allows for addressing any potential conditionality and any violations of the preferential and utility independence.

The next step is to extract nurses’ preferences using pairwise comparisons, verbal discussions, and case-based scenarios. Lottery questions are created to extract nurses’ values for each attribute to model their individual preferences in a classification tree. Additionally, rather than using crisp values, fuzzy AHP [92,93,94,95,96] is used to determine a range in which a nurse decision-maker is willing to give up the probabilistic best payoff as well as the worst, also known as the Certainty Equivalent (CE).

Subsequently, to obtain the parameters needed for the aggregation of the utilities, another set of questions is constructed to determine the tradeoff values for nurses. The attribute tradeoff is a measure of how much the DM is willing to give up one attribute to gain a specific amount on another attribute [97]. In the healthcare scenario discussed here, the tradeoff represents how much a nurse is willing to let an attribute get closer to a critical value before he/she thinks that this attribute is as critical as a value of another attribute.

Once the values obtained through interviews are validated with the regression trees, the dynamic MAUT models are constructed. Characterization of the enhanced MAUT definition that considers changes of the attributes over time is presented in Equation (1).

(1) $$U_{ik}\left(X_{i}\left(t\right)\right)=\;\overset{2}{\underset{j=1}{\sum }}\left[U_{ijk}\left(X_{i}\left(t\right)\right)\right]=\;\overset{2}{\underset{j=1}{\sum }}\left[(A_{ijk}-\;B_{ijk}*\;e^{\left(\frac{-X_{i}\;\left(t\right)}{RT_{ijk}}\right)})*\;y_{ij}\left(t\right)\right]$$

where *x_i_*(*t*) is the actual value of an attribute *i* at time *t*, *RT_ijk_* represents the risk tolerance for attribute *i* at subset level *j*, for nurse *k*; *A_ijk_* and *B_ijk_* represent scaling parameters as given by Equations (2)–(4). The variable *y_ij_*(*t*) is a binary value used to account for the fact that some attributes are non-monotonic. The values for each of these attributes can only belong to one subset for each instance in time. For example, a patient can only have a pulse that is either less or greater than or equal to the preferred value but not both conditions concurrently. Hence, the binary variable is described by Equations (5) and (6).

(2) $$A_{ijk}=\frac{e^{\left(\frac{-Min\left(x_{ij}\right)}{RT_{ijk}}\right)}}{\left[e^{\left(\frac{-Min\left(x_{ij}\right)}{RT_{ijk}}\right)}-e^{\left(\frac{-Max\left(x_{ij}\right)}{RT_{ijk}}\right)}\right]}$$

(3) $$B_{ijk}=\frac{1}{\left[e^{\left(\frac{-Min\left(x_{ij}\right)}{RT_{ijk}}\right)}-e^{\left(\frac{-Max\left(x_{ij}\right)}{RT_{ijk}}\right)}\right]}$$

(4) $$RT_{ijk}=\frac{-CE_{ijk}}{ln\left(\frac{-0.5U_{ijk}\left(Max\left(x_{ij}\right)\right)-0.5U_{ijk}\left(Min\left(x_{ij}\right)\right)+A_{ijk}}{B_{ijk}}\right)}$$

(5) $$y_{ij}\;\left(t\right)=\;\{\begin{array}{c} 1\\ 0 \end{array}\;\;\;\;\;\begin{array}{c} if\;sub-attribute\;j\;is\;present\;at\;time\;t\\ otherwise \end{array}$$

(6) $$\overset{2}{\underset{j=1}{\sum }}y_{ij}\left(t\right)=1\forall i$$

where *RT_ijk_* = Risk tolerance for attribute *i* at subset level *j*, for nurse *k*; *Min* (*x_ij_*) = Minimum value for attribute *i* at subset level *j* across all alternatives; Max (*x_ij_*) = Maximum value for attribute i at subset level *j* across all alternatives; *CE_ijk_* = Certainty equivalent for attribute *i* at subset level *j*, for nurse *k*. The Aggregated Utility for each nurse, *k*, uses the multiplicative form then takes into account the fact that there are multiple DMs and is expressed as:

(7) $$U_{k}\left(x\left(t\right)\right)=\frac{1}{K_{k}}\left[\overset{q}{\underset{i=1}{\prod }}\left(K_{k}k_{ik}U_{ik}\left(x_{i}\left(t\right)\right)+1\right)-1\right]$$

where *U_k_*(*x*) = the multi-attribute utility of x for nurse k; *xij*(*t*) = the performance level of attribute *ij* at time *t*; *U_ik_*(*xij*(*t*)) = the single attribute utility for attribute *ij*, for nurse *k* at time *t*; *i* = 1, 2, 3,…, *q* attributes; *k_ik_* = attribute-scaling parameter for attribute *i*, for nurse *k*; *K_k_* = normalizing constant for nurse *k*, given by:

(8) $$1+K_{k}=\overset{q}{\underset{i=1}{\prod }}\left(1+K_{k}k_{ik}\right)$$

Finally, nurses’ utilities are aggregated to minimize the differences between nurses. This is done because it is not reasonable for a patient’s priority to be dependent exclusively on the nurse doing the triage, even though this might be the case. Vector scaling was chosen to explore the possibility of using an aggregation method that did not involve a person’s judgment. To the best of the researcher’s knowledge, combining vector scaling with MAUT has never been performed.

Vector scaling uses *L_p_* Norm to normalize the data where the function is defined according to the number assigned to *p*. *L_p_* Norms measure the lengths of vectors while *L_p_* metrics measure the distance between points [98]. Usually, after normalizing the data with *L_p_* Norm, the *L_p_* metric is used to obtain the best alternative. In a study conducted by Powdrell (2003), he concluded that applying the *L*_1_ Norm (*L_p_* Norm when *p* = 1) to several of the ranking algorithms “increases the accuracy of the algorithm and helps eliminate ties in the rankings.” For this reason, the L_1_ Norm combined with *L*_1_ metric is proposed. The generalized *L_p_* Norm of a vector is given by:

(9) $$\|x{\|}_{p}={\left[\overset{n}{\underset{j=1}{\sum }}|x_{j}{|}^{p}\right]}^{1/p}$$

It should be noted that when *p* = 1, the absolute value is taken. Additionally, in the current problem, the values that are being considered are the utilities calculated from each nurse for each patient. Thus, the L_1_ Norm for the current problem is expressed as:

(10) $$\|x_{k}{\|}_{1}=\overset{m}{\underset{l=1}{\sum }}|U_{kl}\left(x\left(t\right)\right){|}^{\;}\forall k$$

where l is the patient number (*l* = 1, 2... *m*), and k is the nurse number. Once the *L*_1_ Norm is calculated for each nurse, then the data do not need to be normalized. The vector normalization is given by Equation (11).

(11) $$r_{kl}=\frac{U_{kl}\left(x\left(t\right)\right)}{\|x_{k}{\|}_{1}}$$

where *r_k_*_l_ = normalized value of utility of patient *l* for nurse *k* and *U_kl_*(*x*(*t*)) = utility of patient *l* for nurse *k*. After the data have been normalized, the distances between the current value and the maximum value can be calculated with the *L_p_* metric. The *L_p_* metric between two vectors *x*, *y*, where *x*, *y* ∈ *Rn* is given by:

(12) $$L_{p}={\left[\overset{n}{\underset{j=1}{\sum }}|x_{j}-y_{j}{|}^{p}\right]}^{1/p}$$

Hence, the *L*_1_ metric between two vectors is expressed as:

(13) $$L_{1}=\overset{n}{\underset{j=1}{\sum }}|x_{j}-y_{j}{|}^{}$$

If one of these points (i.e., *y_j_*) is the ideal solution, then the distance represents how close each alternative is to the ideal [99]. In the current problem, the ideal solution represents the patient with the maximum utility value for each nurse. Hence, the *L*_1_ metric represents the sum of the distances of a patient to the patients with the highest utility for the different nurses. For this reason, the smaller the distance, the closer an alternative is to the ideal solution.

In theory, if a patient has the maximum utility value for all the nurses, then this patient has the highest priority without any uncertainty. However, since nurses value different attributes differently, and hence, make decisions differently, in reality, this might not be the case. In the current problem, the highest assigned value from each nurse to the patients, denoted by *R_k_*, and the *L*_1_ Norm for each patient between his/her utility value and the highest assigned value for each nurse, is expressed as:

(14) $$R_{k}=\|x{\|}_{\infty }=max(\left|r_{k1}\left|,\ldots ,\right|r_{km}\right|)\;\forall k$$

(15) $$L_{1l}=\overset{n}{\underset{k=1}{\sum }}|r_{kl}-R_{k}{|}^{}\;\forall l$$

Advantages of using MAUT over some of the other triage support systems include: it eliminates clustering to provide an absolute prioritization of patients; MAUT is a multiplicative approach and accounts for nurse’s preferences, while the DGP algorithm does not account for the decision maker’s preference; as with eTriage, the MAUT model can incorporate nurse preferences using computer learning and consider previous overrides and past decisions; however, MAUT offers the additional benefit of prioritizing between patients assigned to the same triage level.

**Figure 5:**
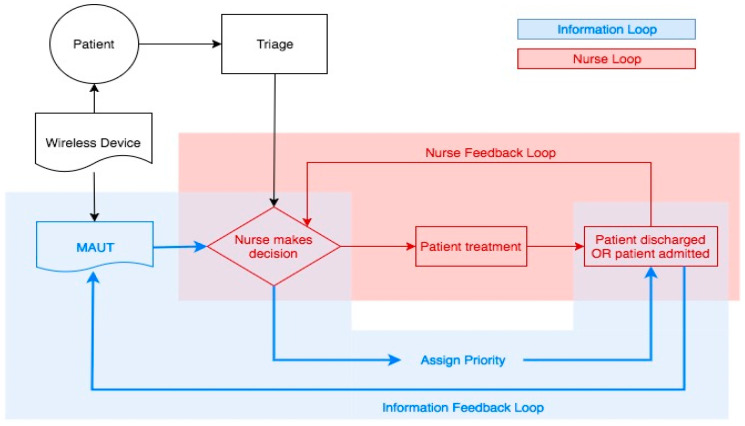
Proposed Nurse and Information Feedback Loops.

The proposed conceptual model, presented in Figure 5, incorporates a feedback loop that relays information to nurses in addition to feeding into a MAUT algorithm. Closing the information loop for nurses can help to fine tune knowledge, intuition, and experience. Using information from the patient’s visit outcome can help the MAUT decision support system reflect user preferences and improve prioritization suggestions. Figure 5 highlights these two feedback loops and how wireless wearable devices and MAUT can facilitate nurse decision making.

The feedback system’s two goals include: (1) create a mathematical MAUT model that is as close as possible to the nurse decision; (2) make both the nurse decision and the mathematical model reflect the final outcome as accurately as possible. The first goal is reflected in the conceptual model by the intersection of the two loops. The MAUT model is dependent on the nurse’s decisions and the final outcomes of patient visits. Moreover, it is constantly being updated by receiving constant information from patients via wireless monitoring devices.

The second goal is reflected by the two feedback loops: the red loop that fine-tunes the nurse decision-making and the blue loop that refines the mathematical model. Ultimately the simultaneous feedback from the two loops, in conjunction with data obtained from continuous vital signs monitoring, will help prioritize patients using mathematical models and nurse preference.

The MAUT algorithms will incorporate nurse preference and information from continuous vital signs monitoring to rank and monitor patients. The proposed model will use an adaptive system based on the feedback loops and will periodically compare the nurses’ values versus the values it suggested. If the values are significantly different, the algorithm will then evaluate those instances and use classification trees to generate the new values for the utility models. This will allow the model to evolve as the nurses’ preferences change.

Additionally, since the decision of patient criticality is not going to be based on the status of the patient in one instant in time but rather considering continuous vital signs information, statistical process control (SPC) will be used to monitor the overall status of patients and to discover any trends in patient criticality. SPC will be applied to each individual attribute to develop an emergency alert system, informing nurses of any critical changes in patient status.

The coupling of wireless wearable devices and decision theory, such as MAUT, addresses the previously mentioned four shortcomings of current triage systems in the US, offering an automated, quantified, and structured decision system.

## 4. Discussion

### 4.1. Feasibility of the Proposed Clinical Decision Support Model

The literature review offered insight and valuable information that can be considered while moving forward with the proposed conceptual model.

#### 4.1.1. Hospital Liability

Curtis et al. emphasize that a hospital assumes additional liability to respond to vital signs abnormality once they continuously monitor a patient. In the case where a hospital is monitoring a patient but does not respond to a critically abnormal vital sign, there may be grounds for litigation, “even if the risk of not detecting an event in this system was the same as if the patient had not been monitored, as in the current standard of care.” [41]. An additional consideration of liability is if the wearable device fails to detect a patient’s abnormal vital signs.

#### 4.1.2. Quality of Data

The information obtained from continuously monitoring vital signs must be accurate with minimal noise and missing data [100]. The quality of data can be affected by the wireless device’s reliability and patients wearing devices incorrectly.

Additionally, the system should incorporate a procedure to address false positives. For example, the SMART system resulted in a high number of false alarms and therefore incorporated an operator to act as an intermediator to filter information from the alarms [41]. Ultimately the accuracy and reliability of the proposed MAUT system will depend on the quality of data that feeds into it; therefore, the data must be accurate.

#### 4.1.3. Response to Data and Workload Impacts

While the proposed system uses vital signs monitoring to prioritize patients, another consideration is to use the system to incorporate additional interventions. For example, information from a patient’s vital signs can be used to signal medication administration, positional changes, comfort measures, notification of physician, or other escalation procedures [10]. While these interventions, in addition to the proposed model, present an opportunity to improve patient care, they also directly impact ED nurse workload. Using wearable devices to monitor vital signs continuously can eliminate data entry into the electronic medical record by automatically feeding data from the device into the computer record. However, while this may reduce the workload impacts associated with collecting patient vital signs, the proposed model impacts nurse workload in other ways.

The quality of patient care can be negatively affected by work interruptions [10]. This highlights the importance of considering the impact of exposing nurses to additional information in the form of continuous vital signs data and MAUT prioritization outputs. Additionally, an ED’s complex environment can make it even more difficult for nurses to be alerted to warning signals [101]. A nurse’s duties in an ED are complicated by factors such as administrative requirements, computer data entry, phone calls, pagers, conversations, and monitoring alarms with both auditory and visual outputs of patient physiological status for a large number of patients [101]. Signal detection theory could help understand how nurses identify the important signals while being exposed to the complex environment of interfering information and noise from the ED [101].

Using signal detection theory, a nurse classifies a signal as either a hit, miss, false alarm, or correct rejection. A "hit" indicates that the nurse correctly identifies the signal and a "correct rejection" means the nurse correctly identified the noise [101]. A "miss" means the nurse did not identify the signal when she/he should have, and a "false alarm" indicates the nurse identified a signal when there was none [101]. The nurse’s goal is to maximize hits and correct rejections while minimizing the number of misses and false alarms. Exposing nurses to additional information with continuous vital signs monitoring and prioritization recommendations can increase the background noise, therefore complicating this signal detection decision-making process. The result of this may be detrimental because nurses will be less sensitive to the occasional alarm for an urgent condition if they are continuously exposed to alarms that sound for negligible reasons [102]. This reduced sensitivity would lead to an increased number of misses. On the opposite end of the spectrum, Jerison and Pickett found that oversensitivity will lead to frequent false alarms [103].

To be beneficial, the proposed model must not negatively impact the nurse’s sensitivity to signals in the ED by overstimulating nurses with additional noise and information. Various factors can help control sensitivity to signals, such as reducing ambient sound levels and developing distinct signal notifications [104]. Ultimately, further research must explore how nurses’ decision-making abilities are impacted by additional noise created by the proposed patient monitoring and prioritization method.

#### 4.1.4. Technology Acceptance

Claudio, Velazquez, Bravo-Llerena, Okudan, and Freivalds (2015) [105] studied the perceived usefulness and ease of use of wearable sensor-based systems in EDs. They found that patients and nurses had positive reactions to the perceived usefulness and ease of use of a wireless wearable sensor-based system at two EDs. In fact, patients’ perceptions of the ease of use were more favorable and less variable than nurses’ perceptions.

## 5. Conclusions

The coupling of wearable devices with decision theory to continuously monitor patients in the ED can help prioritize patients and address shortcomings in current ESI methods and triage systems. Four shortcomings that the proposed use of wearable devices and decision theory can address include (1) patient monitoring [13]; (2) subjectivity in triage assessment and uncertainty [14]; (3) prioritization on an FCFS basis within a group; (4) lack of feedback for nurses on their prioritization decisions.

Many systems regarding patient prioritization in the ED have been developed and researched. However, none of these applications address all four of the shortcomings mentioned above, highlighting the opportunities to conduct further research to determine if the application of the proposed model in an ED setting can help with nurse decision-making and improve patient care quality and, therefore, the outcome of ED visits.

Future research should look further into the implementation approach for the proposed decision support model, which will help gain user acceptance and realize all the model’s benefits. An implementation plan and a usability test plan to integrate the system into ED operations for testing and research are part of this research’s future steps. Likewise, evaluating the impacts on nurses and patients should take part in the usability and implementation plan. Future research would also contemplate the analysis of how the multimodal data stream wearable devices enhance decision-making and the validation of the clinical decision-making support system.

## Figures and Tables

**Table 1:** Summary of wearable devices.

Device	Manufacturer	Status	Type
1. MIThril (LiveNet) [55]	MIT	Unreleased/Project Finished	Wearable Platform/Package-Multiuse
2. AMON [56]	EUIST	Unreleased/Project Finished	Wrist-worn Multisensor
3. LifeGuard [57]	Stanford/NASA	Unreleased/Project Finished	Custom Chest Device and commercial sensors
4. MyHEART [58]	EUIST	Unreleased/Project Finished	Smart Textiles
5. WEALTHY [59]	EUIST	Unreleased/Project Finished	Smart Textiles
6. MagIC [60]	University of Milan	Unreleased/Project Finished	Smart Textiles/Sensorized Vest
7. MERMOTH [61]	EUIST	Unreleased/Project Finished	Smart Textiles
8. CodeBlue [62]	Harvard	Architecture	Architecture for Ad Hoc Sensor networks
9. WSN u-Healthcare [63]	Dongseo University	Routing Protocol	Routing protocol for data from wearables
10. Human++ [64]	IMEC	Commercial	ULP circuits, custom hardware, commercial wearable marked
11. HeartToGo [65]	University of Pittsburgh	Architecture	Architecture uses Alive Tec chest band
12. AUDABE [66]	Dept. Medical Physics Greece	Unreleased/Project Finished	Mask/Glove for emotional state detection in stressful environments
13. LifeShirt [67]	Vivometrics	Commercial	Shirt for all day monitoring
14. BioHarness [68]	Medtronic	Commercial	Shirt, strap, harness for performance monitoring
15. ViSi Mobile [69]	Sotera Wireless	Commercial	Wrist cuff, wires, stick-on electrodes
16. EquiVital (LifeMonitor) [70]	Hidalgo	Commercial	Strap, extra peripherals
17. Aingeal [71,72]	Intelesens	Commercial	Device, electrodes
18. VitalSens VS100 [73]	Intelesens	Unreleased/Project Finished	
19. QUASAR [74]	Wearable Sensing	Commercial	Headset, EEG
20. Alive BTH&Activity [75]	Alive Technologies	Commercial	Electrodes, bodypack
21. BodyGuardian Heart [76]	Preventive	Commercial	Patch and module
22. CareTaker 4 [77]	CareTaker Medical	Commercial	Wrist cuff and finger cuff

**Table 2:** Device capabilities.

Device Name	Heart Rate	Pulse Oxi- MeterSpO2	Systolic and Diastolic Blood Pressure	Blood Pressure Automatic Measure	Telemetry	Respiration Rate	Pain Level	Temperature	Activity
1. MIThril [55]	x	x	x	Assumed	ECG, EMG	x		x	
2. AMON [56]	x	x	x	x	ECG			x	x
3. LifeGuard [57]	x	x	x	x	ECG	x		x	x
4. MyHEART [58]	x				ECG	x			x
5. WEALTHY [59]	x				ECG, EMG	x		x	x
6. MagIC [60]					ECG	x		x	
7. MERMOTH [61]					ECG	x		x	x
8. CodeBlue [62]		x	x	Assumed	ECG				x
9. u-Healthcare [63]									
10. Human++ [64]									
11. HeartToGo [65]									
12. AUDABE [66]					ECG, EMG	x			
13. LifeShirt [67]									
14. BioHarness [68]					ECG	x		x	x
15. ViSi Mobile [69]	x	x	x	cNIBP		x		x	
16. EquiVital [70]	x	x			ECG	x		x	x
17. Aingeal [71,72]	x				ECG	x		x	
18. VitalSens VS100 [73]									
19. QUASAR [74]					ECG			x	x
20. Alive BTH& Activity [75]	x				ECG				x
21. BodyGuardianHeart [76]	x				ECG				
22. CareTaker 4 [77]	x	x	x	cNIBP		x	Can add	x	
